# Identification of a Novel *NOG* Missense Mutation in a Chinese Family With Symphalangism and Tarsal Coalitions

**DOI:** 10.3389/fgene.2019.00353

**Published:** 2019-04-18

**Authors:** Jing Xiong, Wei Tu, Yifei Yan, Kai Xiao, Yanyi Yao, Shouxin Li, Liu Yang, Min Zhou, Yang Liu, Jin Hu, Feng Zhu

**Affiliations:** ^1^Department of Nephrology, Union Hospital, Tongji Medical College, Huazhong University of Science and Technology, Wuhan, China; ^2^Department of Rheumatology and Immunology, Tongji Hospital, Tongji Medical College, Huazhong University of Science and Technology, Wuhan, China; ^3^Département de Biochimie et Médecine Moléculaire, Institut de Recherche en Immunologie et en Cancérologie, Université de Montréal, Montreal, QC, Canada; ^4^Department of Foot and Ankle Surgery, Wuhan Puai Hospital Affiliated to Tongji Medical College, Huazhong University of Science and Technology, Wuhan, China; ^5^Medical Genetic Center, Maternal and Child Health Hospital of Hubei Province, Wuhan, China; ^6^Reproductive Medicine Center, Tongji Hospital, Tongji Medical College, Huazhong University of Science and Technology, Wuhan, China; ^7^Department of Respiratory and Critical Care Medicine, Tongji Hospital, Tongji Medical College, Huazhong University of Science and Technology, Wuhan, China; ^8^Aegicare (Shenzhen) Technology Co., Ltd., Shenzhen, China; ^9^Department of Otolaryngology, Head and Neck Surgery, Tongji Hospital, Tongji Medical College, Huazhong University of Science and Technology, Wuhan, China; ^10^Clinic Center of Human Gene Research, Union Hospital, Tongji Medical College, Huazhong University of Science and Technology, Wuhan, China; ^11^Department of Cardiology, Union Hospital, Tongji Medical College, Huazhong University of Science and Technology, Wuhan, China

**Keywords:** proximal symphalangism, tarsal coalition, *NOG* gene, mutation, whole exome sequencing

## Abstract

**Background:**

Proximal symphalangism (SYM1) is a rare genetic bone disorder characterized by the fusion of proximal interphalangeal joints in the hands and feet. Genetic studies have identified two genes underlying SYM1 as the noggin (NOG) and the growth differentiation factor 5 (GDF5).

**Case Report:**

In the present report, a 43-year-old gravida at 11 weeks of gestation was referred for evaluation of abnormal fusions of the joints. In the initial diagnosis, physical examination was undertaken. However, traditional radiological examination was not applied due to the need to protect the fetus, making diagnosis results inefficient to determine the exact disease affecting the proband. To acquire alternative clinical evidences, we conducted radiological examinations on two other affected family members. The radiological examination revealed that they carried the symphalangism accompanied with tarsal coalition, a very rare manifestation of SYM1. A combination of whole exome sequencing (WES) and Sanger sequencing revealed a novel heterozygous missense mutation (c.163G > T; p.Asp55Tyr) in the NOG gene, which could be associated with the observed pathogenic SYM1 in the studied family. The p.Asp55Tyr mutation co-segregated with SYM1 through the affected and unaffected family members. *In silico* structural modeling of the p.Asp55Tyr mutation showed that it abolishes the interaction with the Arg167 residue and causes a change in the electrostatic potential profile of the type II binding site of the noggin protein.

**Conclusion:**

Our findings indicate that the genetic test based on WES can be useful in diagnosing SYM1 patients, with particular advantages in preventing the fetus from contacting harmful X-ray through the traditional radiography. The novel pathogenic mutation identified would further expand our understanding of the mutation spectrum of *NOG* in association with SYM1 disease and provide a guidance on how to determine whether the fetus is affected by SYM1 through the prenatal diagnosis.

## Introduction

Proximal symphalangism (MIM#185800, SYM1) is a rare autosomal dominant bone disorder with principal features of variable fusions of the proximal interphalangeal joints of fingers, toes, carpus, and tarsus, as well as conductive hearing loss in some cases (Strasburger et al., 1965). Previous genetic studies have uncovered that two genes responsible for the SYM1 are the *NOG* gene (MIM#602991) (Gong et al., 1999) and the *GDF5* gene (MIM#601146) (Wang et al., 2006). The *NOG* gene, located on chromosome region 17q22, encodes the Bone Morphogenetic Protein (BMP) antagonist, noggin, which is 232 amino acids in length and essential for normal bone and joint development in humans and mice (Potti et al., 2011). One study on NOG-null mouse demonstrated that *NOG* inactivation caused excess BMP activity, overgrowth of cartilage and failure to initiate joint formation (Pang et al., 2015). According to the latest statistics from the Human Gene Mutation Database^1^, 62 *NOG* functional mutations are considered to be responsible for SYM1; in addition, such mutations are also responsible for some other similar syndromes including brachydactyly type B2 (MIM#611377, BDB2), multiple synostoses syndrome 1 (MIM#186500, SYNS1), stapes ankylosis with broad thumb and toes (MIM#184460, SABTT) and tarsal-carpal coalition syndrome (MIM#186570, TCC). The purpose of this study was to investigate the disease-related genes in a three generation Chinese pedigree with SYM1 and guide prenatal diagnosis of the proband. Given that whole exome sequencing (WES) is a powerful and cost-effective tool for genetic testing, we performed WES on the proband. Upon carefully analysis of the clinical impacts of all the mutations identified, we confirmed that novel missense mutation c.163G > T (p.Asp55Tyr) in *NOG* gene (reference NM_005450) was the pathogenic mutation in this family. The result from our study may provide new insights into the pathogenic mechanism of SYM1.

## Case Report

**FIGURE 1:**
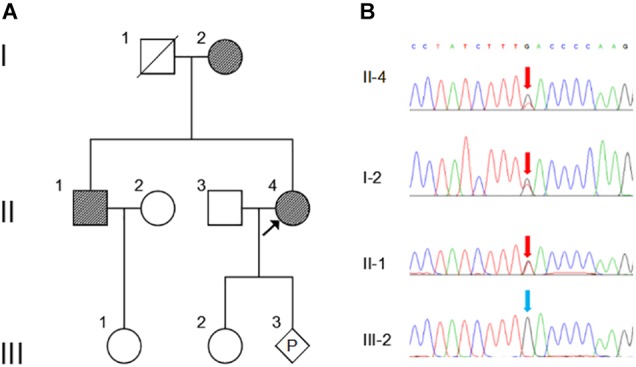
Pedigree and Sanger sequencing chromatogram of c.163G > T (p.Asp55Tyr) mutation in *NOG*. **(A)** Family pedigree. Black arrow indicates the proband, II-4. P, represents pregnancy, III-3. **(B)** The mutation c.163G > T in *NOG* is identified in II-4 (proband), I-2 and II-2 (red arrows). Blue arrow indicates wild type in III-2. The reference sequence NM_005450 of *NOG* is used.

**FIGURE 2:**
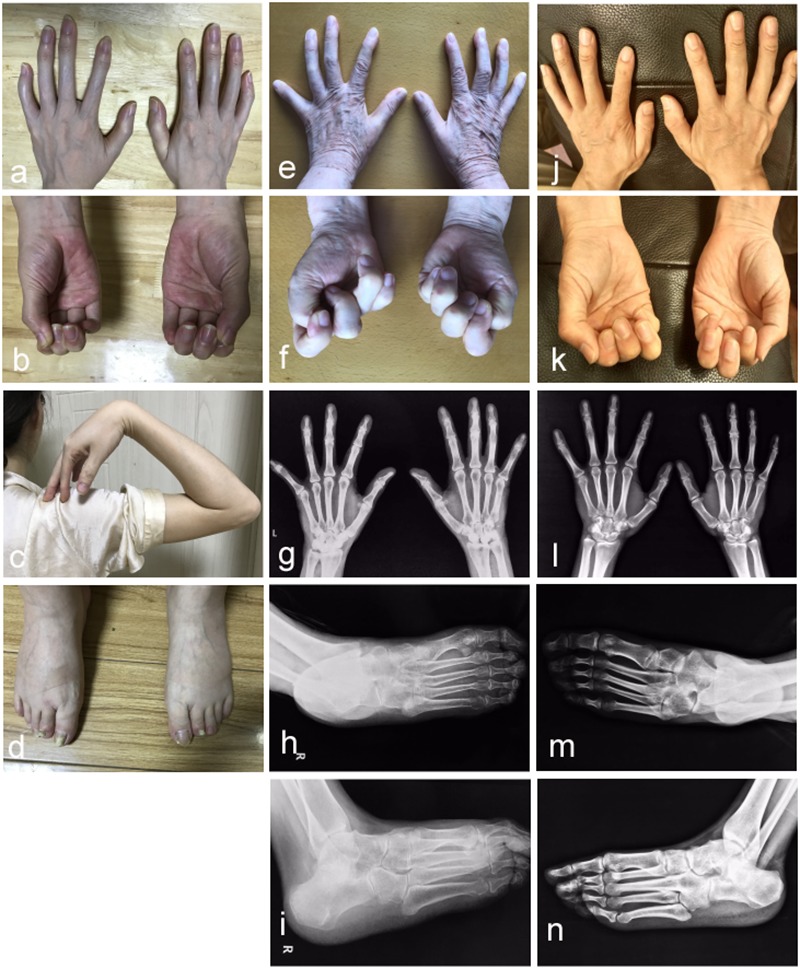
Photographic features of hands and feet in the affected individuals. Abnormal joint osseous fusion in fingers and difficulties in making fists were seen in all of patients II-4 **(a,b)**, I-2 **(e,f)** and II-1 **(j,k)**. For patient II-4, the motion of the elbow joint is normal **(c)**, and a little short phalange of second toe is noticed in both her feet **(d)**. Radiographs of the hands reveal that patient I-2 **(g)** and II-1 **(l)** have proximal symphalangism in hands. Radiographs of right feet in anteroposterior and oblique view show that patient I-2 **(h,i)** presents proximal symphalangism in toes and tarsal coalitions, while patient II-1 **(m,n)** has both proximal and distal symphalangism in toes and tarsal coalition.

In the studied family, a 43-year-old gravida at 11 weeks of gestation (the proband, II-4), her mother (I-2) and sibling (II-1) were presented with abnormal fusions of joints (Figure 1A). The clinical features of hands and feet are listed and provided in Table 1. In the initial observation on the fingers of the proband (II-4), it was noticed that both thumbs were normal (Figure 2a); however, finger 2–5 lacked creases on flexor and extensor surfaces of the interphalangeal joints (Figure 2a), and was not able to flex (Figure 2b). Further examination on elbow joints demonstrated that she had normal abilities to move them and was able to touch her shoulder with hands (Figure 2c). On checking both feet of the proband (Figure 2d), we found abnormalities similar to the fingers: (i) both thumbs were normal; (ii) both toe 2–4 lacked creases on flexor and extensor surfaces of interphalangeal joints; and (iii) the symptomatic toes were not able to flex. Furthermore, a little short phalange of second toe was noticed in both feet (Figure 2d). Given that the proband was a gravida at 11 weeks of gestation, X-ray examinations that could enable uncovering of the status of the osseous fusion were not applied to her. Patient I-2 and II-1 had similar symptoms as the proband, therefore, detailed examinations including X-ray examinations were supposed to provide alternative evidences for the proband. Like proband, both patients I-2 and II-1 had normal elbows. For patient I-2, proximal interphalangeal joint osseous fusion of left finger 4–5 and right finger 4–5 were observed (Figure 2e–g), but tarsal-carpal coalition was not found. Foot radiographs revealed that patient I-2 had proximal interphalangeal joint osseous fusion of left toe 3–5 and right toe 2–4 (Figure 2h), and she had bilateral talonavicular coalition and talocalcaneal coalition of feet (Figure 2h,i). For patient II-1, both observation and radiographs showed proximal symphalangism of left fingers 3–5 and right fingers 4–5 (Figure 2i–k). Proximal symphalangism was evident in right toe 3–4, while distal interphalangeal joint osseous fusions were observed on left toe 2–5 (Figure 2m,n). Furthermore, fusion of multiple tarsal bones was found in patient II-1 (Figure 2m,n), that is, bilateral talonavicular coalition, bilateral talocalcaneal coalition, coalition of the cuboid bone and the third cuneiform bone on left side as well as coalition of the third cuneiform bone and the third metatarsal bone on the left side. Pure-tone audiometry (125 Hz–8 kHz) showed normal hearing thresholds in all the members.

**Table 1:** Clinical features of the patients in pedigree.

Patient ID	Right fingers	Left fingers	Right toes	Left toes	Left foot	Right foot
I-2	4–5 fp	4–5 fp	2–4 fp	3–5 fp	tn and tc	tn and tc
II-1	4–5 fp	3–5 fp	3–4 fp	2–5 dp	tn, tc, cc, and cm	tn and tc
II-4	2–5 fp	2–5 fp and 5dp	2–4 fp	2–4 fp and 2ds	NA	NA
III-2	no fp	no fp	no fp	no fp	none of tn, tc, cc or cm	neither tn or tc

*fp, a fused proximal joint; ds, a little short phalange; dp, a fused distal joint; tn, talonavicular coalition; tc, talocalcaneal coalition; cc, the fusion between cuboid bone and the third cuneiform bone; cm, the fusion between the third cuneiform bone and the third metatarsal bone; NA, no data.*

WES was performed on the proband and total of 136,155 variants, including 122,267 single nucleotide variants, 13,882 indels (deletions and insertions, <50 bp) and 6 copy number variations were identified by WES, followed by annotation, filtration and prioritization steps to obtain the variants causing symptoms in the studied family (Supplementary Table 1). The results indicate that *NOG* is the most likely candidate gene. Pathogenicity analysis was performed to associate the identified *NOG* mutation with the syndromes of the studied Chinese family. Among all identified mutations of the proband, only one heterozygous G-to-T transversion c.163G > T at genomic position 54,671,747 of chromosome 17 was located in *NOG*, resulting in a substitution of aspartic acid to tyrosine at codon 55 (p.Asp55Tyr). Through literature search, we did not find the p.Asp55Tyr recorded in any database or on any previous publication. The evaluation of possible functional impacts revealed that p.Asp55Tyr was classified as a damaging mutation by multiple missense prediction tools (Table 2). Comparative amino acid sequence alignment of NOG protein across different species in mammals revealed that the novel p.Asp55Tyr mutation occurred at highly conserved position (Supplementary Figure S1). Furthermore, Sanger sequencing analysis identified p.Asp55Tyr in all the three affected family members (I-2, II-1, and II-4) while it was absent in the unaffected member (III-2) (Figure 1B). Therefore, p.Asp55Tyr co-segregated with the symptoms in the studied family. Additionally, we found that this variant was absent in 200 normal controls, suggesting that p.Asp55Tyr did not represent a rare polymorphism, but was a pathogenic mutation within this Chinese family.

**Table 2:** Predicted effect of the c.163G > T (p.Asp55Tyr) on noggin protein structure by multiple algorithms.

Algorithm	Predicted effect	Score
SIFT	Deleterious	0.005
MutationTaster	Disease causing	0.818
LRT	Deleterious	0.8932
Fathmm	Damaging	0.9839

*SIFT, Sorting Intolerant From Tolerant (Ng and Henikoff, 2003); MutationTaster (Schwarz et al., 2010); LRT, Likelihood Ratio Test (Chun and Fay, 2009); Fathmm, Functional Analysis through Hidden Markov Models (Shihab et al., 2013).*

**FIGURE 3:**
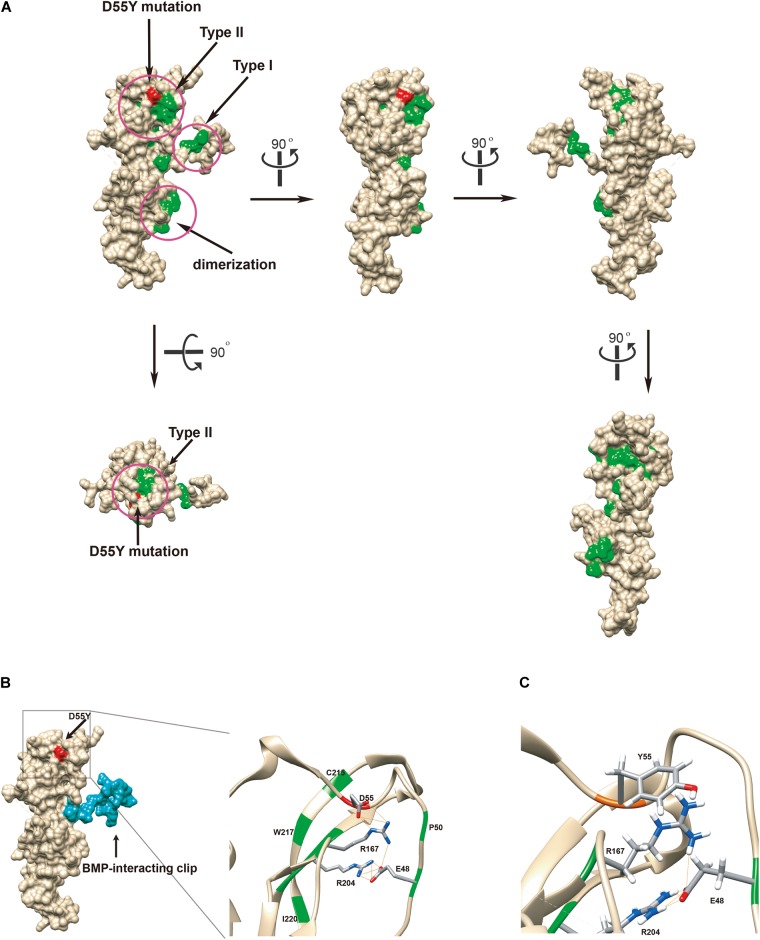
Functional *in silico* docking analysis of the p.Asp55Tyr mutation in the *NOG* gene. **(A)** The disease-associated mutational hotspots on noggin were mapped to show that p.Asp55Tyr is located within the type II receptor binding region. Known mutations are colored green and Asp55 is in red on the Coulombic surface of the protein. Three regions that were previously established are circled. Snapshots of noggin from different point views were taken by rotating the protein 90° at a time. **(B)** Molecular surface view of noggin is derived from a crystal structure (1M4U). The binding ligand, BMP-14, was removed for clear visualization of noggin. The BMP-interacting clip is colored blue. The location of the mutated residue, Asp55Tyr, is colored red. The type II receptor binding site, where Asp55 resides, is enlarged to show its special arrangement. Among all known mutations, only four residues are hydrogen-bonded (lines colored orange) via side chains to form a network: Glu48, Asp55, Arg167, and Arg204 (the EDRR-tetrad). Asp55 preferentially interacts with Arg167. All residues known to be mutated in diseases are colored green. Detected hydrogen bonds are indicated by lines in orange. **(C)** *In silico* generated conformation of the EYRR-tetrad that harbors the p.Asp55Tyr mutation. All three hydrogen bonds are abolished between residue 55 and Arg167. D55Y, Asp55Tyr; C215, Cys215; W217, Trp217; I220, Ile220; D55, Asp55; R167, Arg167; R204, Arg204; E48, Glu48; P50, Pro50; Y55, Tyr55; E48, Glu48.

The crystal structure of noggin protein (Protein Data Bank accession code 1M4U) was used to visualize the spatial arrangement of amino acids around the mutated residue. Modeling was performed in UCSF Chimera as stipulated in the previously established procedures (Burger et al., 2009). Setting of all the parameters is described in Supplementary Materials. To better elucidate the mechanism by which the loss of function occurs, we mapped the identified p.Asp55Tyr (red) and all mutations previously reported in literature (green) to the structure (Figure 3A) (Usami et al., 2012). The disease-associated mutations are concentrated in three regions as previously reported: type I and type II receptor binding domains and the dimerization domain (Figure 3A, in circles) (Lehmann et al., 2007). The aspartate residue (Asp55) is located at a distance from the BMP binding-site called the type I receptor-binding clip (Figure 3B left panel, colored blue) and hence, it is unlikely that this mutation directly affects noggin’s binding affinity to type I site of BMP. The p.Asp55Tyr mutation reported in this study is located within the type II receptor binding region, surrounded by a group of known disease-associated mutations forming a mutational “hotspot” in the noggin protein. Closer look at this mutational hotspot reveals that the aspartate side chain strongly interacts with the positively charged Arg167 residue by hydrogen bonding (Figure 3B right panel). In fact, among all the known mutations within the type II binding region, only four amino acid residues Glu48, Asp55, Arg167, and Arg204 were found to strongly interact with each other via hydrogen bonds between their side chains. Asp55 interacts with the Arg167 by three hydrogen bonds, Glu48 interacts with the Arg204 with four hydrogen bonds, and Tyr167 interacts with Glu48 with one hydrogen bond (Figure 3B). These four amino acid residues, two being positively and two being negatively charged, form a tight cluster and are very likely to hold the elbow-shaped BMP-interacting arm in position.

We replaced the aspartic acid residue with a tyrosine and computed the local structure using molecular modeling. Using the most probable conformation of the tyrosine residue (36.63% probability), we added hydrogens and analyzed the hydrogen bonds. All the three hydrogen bonds that were visualized between the aspartic acid and tyrosine 167 were abolished (Figure 3C); this indicated that p.Asp55Tyr was indeed a destabilizing mutation of the local protein folding.

## Discussion

In the present study, we described the clinical features of a Chinese family with proximal symphalangism-1A and tarsal coalitions, which are caused by a novel mutation in the *NOG* gene. This finding is consistent with the known *NOG* pathogenicity in proximal symphalangism (Takahashi et al., 2001). Patients in this family had remarkable proximal symphalangism, including bilateral proximal interphalangeal joint osseous fusion of fingers and toes, as well as fusion of multiple tarsal bones. The symptoms of these two rare skeletal anomalies occurring in the same person is a very rare case that has never occurred in many of the cases so far reported (Geelhoed et al., 1969). The mutations of *NOG* is responsible for several phenotypes of bone diseases, so-called *NOG* related symphalangism spectrum disorders (NOG-SSDs), including BDB2, SYNS1, SABTT, TCC, and SYM1. The BDB2 is a subtype of brachydactyly mainly characterized by hypoplasia or aplasia of distal phalanges in combination with distal symphalangism, fusion of carpal/tarsal bones, and partial cutaneous syndactyly (Lehmann et al., 2007). The SYNS1 is characterized by multiple joint fusions, conductive hearing loss and characteristic facial manifestations, including a broad hemicylindrical nose, and thin upper vermillion (Takahashi et al., 2001). The SABTT patients have bilateral conductive hearing loss due to stapes ankylosis, significant hyperopia, broad thumb and broad first toe, but lack evidence of carpal and/or tarsal fusions or symphalangism. The TTC and the SYM1 have similar features of the fusion of carpal and tarsal bones, as well as conductive deaf. Nevertheless, the TTC patients may develop humeroradial fusion, elbow abnormalities, as well as serious deformities of ankle and foot, which might necessitate palliative or surgical intervention (Potti et al., 2011). Therefore, phenotypes of the studied family are most similar to the presentations of SYM1 among all the NOG-SSDs.

Type I and type II membrane bound serine/threonine kinase receptors respond to the secreted TGF-β ligand (ten Dijke et al., 1996; Massague, 2000) and execute important functions in early and late stages of embryonal development, as well as tissue repair in adults (Hogan, 1996; Reddi, 1998; Massague, 2000). The BMP belongs to the TGF-β superfamily that triggers the dimerization of type I and II receptors and activates intracellular SMAD transcription factors (Derynck et al., 1996). Noggin antagonizes BMP signaling by blocking the type I and type II receptor binding sites of the BMP ligand (Groppe et al., 2002). A small peptide at the N-terminus of noggin, called the clip, blocks the type I receptor binding site of BMP, while a larger structure formed by loops and β-sheets at the C-terminus blocks the type II receptor binding site. Mutations in these two regions impair the antagonizing function of noggin, causing the dysregulated activation of the type I and type II receptors, that results to a variety of genetic diseases in bone development.

The reported mutation, p.Asp55Tyr, is indeed part of a hydrogen-bond network formed with three other residues (Glu48, Arg167, and Arg204) that have been previously reported. The p.Glu48Lys mutation was identified to be associated with both SYM1 and BDB2 (Kosaki et al., 2004; Lehmann et al., 2007), the p.Arg167Gly mutation was involved in BDB2 (Lehmann et al., 2007), and the p.Arg204Leu and p.Arg204Gln mutations were related to TCC (Dixon et al., 2001; Das Bhowmik et al., 2018). By identifying the p.Asp55Tyr mutation in SYM1 in this study, we have confirmed that mutations in any one of the amino acids within the tetrad are associated with disease phenotypes. All four amino acids are located in the type II receptor binding region of noggin. It has been suggested that mutations in these key amino acids are the likely causes of local structure changes in the β-sheet domain, leading to local unfolding. In addition, altered charges in the binding site might decrease the affinity of noggin to the type II receptor-binding ligands (Hellmann and Mueller, 2012).

Functional studies had been conducted to elucidate the effects of the p.Arg167Gly mutation, which closely interacts with the Asp55 residue identified in the present study. Though p.Arg167Gly causes BDB2, the mutant protein only seems to be partially compromised in function in micromass assay (25% loss in its ability to inhibit chondrogenesis) (Lehmann et al., 2007). The assay was performed using chicken limb bud cells transduced with an overexpression vector that carries the mutant noggin. Intracellular overexpression of the partially functional noggin mutant might have compensated for its dosage insufficiency, since the *NOG* mutations are mostly halplo-insufficient, rather than dominant-negative in nature (Dixon et al., 2001). As demonstrated in cotransfection experiments (Marcelino et al., 2001), the human disease-causing mutations in *NOG* are hypomorphic alleles that reduce secretion of functional dimeric noggin.

In conclusion, this study reports a novel mutation, c.163G > T (p.Asp55Tyr) of the *NOG* gene in a Chinese family with SYM1 and tarsal coalitions. Indeed, the result from this study confirms the involvement of *NOG* gene in human SYM1 and expand on the current knowledge of *NOG* mutation spectrum associated with SYM1. Furthermore, this study demonstrates that WES is a good alternative approach for traditional clinical examinations that can significantly aid in the diagnosis of complex or special cases, such as, the ones on gravida as reported in this study, thus benefiting them in genetic counseling and prenatal diagnosis.

## Ethics Statement

Written informed consents for the use of DNA samples and permission to publish all images were obtained from the participants or their legal representatives in accordance with the Declaration of Helsinki. This study was approved by the ethics committee of Wuhan Union Hospital in China.

## Author Contributions

WT, KX, SL, MZ, LY, and JH evaluated and cared for the patients. YiY performed the *in silico* structural modeling. YaY and YL carried out genetic testing on the patients. JX and FZ designed the research and wrote the manuscript.

## Conflict of Interest Statement

YL was employed by company Aegicare Technology Co., Ltd. (Shenzhen, China). All other authors declare no competing interests.

## Footnotes

1 http://www.hgmd.cf.ac.uk

## Supplementary Material

FIGURE S1 Conservation analysis of the *NOG* p.Asp55 amino acid residue. The amino acid sequence around position p.Asp55 (in red) of the *NOG* gene is compared among different species in mammals.
