# Supplementary figures and images for: Identification of a Novel NOG Missense Mutation in a Chinese Family With Symphalangism and Tarsal Coalitions
Source: Front Genet. 2019 Apr 18;10:353. doi: 10.3389/fgene.2019.00353 (PMC6499182; doi:10.3389/fgene.2019.00353)

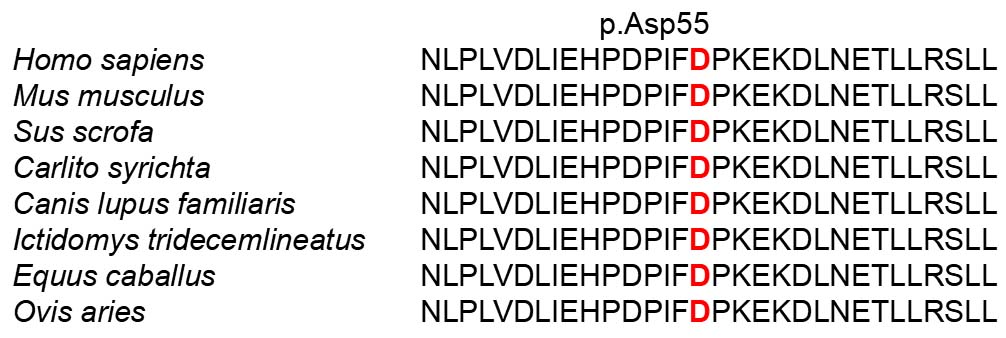

Supplement: FIGURE S1 — Conservation analysis of the NOG p.Asp55 amino acid residue. The amino acid sequence around position p.Asp55 (in red) of the NOG gene is compared among different species in mammals. [file Image_1.JPEG]
