# Supplementary material for: Identification of a Novel NOG Missense Mutation in a Chinese Family With Symphalangism and Tarsal Coalitions
Source: Front Genet. 2019 Apr 18;10:353. doi: 10.3389/fgene.2019.00353 (PMC6499182; doi:10.3389/fgene.2019.00353)
Supplement: Supplementary file 2 [file Table_1.DOC]

**Supplemental Table 1 The number of variants after each prioritization step**

| **Step** | **SNVs** | **indels** | **CNVs** |
| --- | --- | --- | --- |
| Initial | 122,267 | 13,882 | 6 |
| 1. exclude variants in repeats or segmental duplications | 88,171 | 8,264 | 0 |
| 2. exclude variants outside exonic and splicing regions | 23,372 | 946 |  |
| 3. exclude variants with MAF > 0.01 | 694 | 2 |  |
| 4. exclude synonymous variants | 489 | 1 |  |
| 5. exclude non-conservative variants | 489 | 0 |  |
| 6. excluding benign or likely benign variants | 382 |  |  |
| 7. Phenolyzer (term “symphalangism”) | 1 |  |  |
