# Supplementary material for: Identification of a Novel NOG Missense Mutation in a Chinese Family With Symphalangism and Tarsal Coalitions
Source: Front Genet. 2019 Apr 18;10:353. doi: 10.3389/fgene.2019.00353 (PMC6499182; doi:10.3389/fgene.2019.00353)
Supplement: Supplementary file 3 [file Data_Sheet_1.PDF]

Extend Methods

Whole Exome sequencing and Bioinformatics analysis

Whole Exome sequencing was performed on proband (II-4, Figure 1) using Agilent SureSelect Human All Exon V6 Kit on the Illumina HiSeq X Ten platform. According to the manufacturer's protocol, genomic DNA was extracted from whole blood, then sheared by sonication and hybridized for enrichment. After the library was enriched for target regions, sequencing was performed to generate 150 bp paired-end reads. To identify mutations on the proband, sequencing data was analyzed and annotated according to an in-house pipeline. Briefly, raw reads were preprocessed to remove reads with low quality or adapters. Then clean reads were mapped to the human reference genome (GRCh37) using Burrows-Wheelers Aligner (BWA, version 0.7.8-r455) (Li and Durbin 2009). The generated bam file was sorted by SAMtools (Li et al. 2009). Only high-quality alignments were used for the remaining analyses to guarantee variant calling accuracy. Picard (http://sourceforge.net/projects/picard/, version 1.111) was employed to mark duplications resulted from PCR amplification. SAMtools (version 1.0) was performed to call single nucleotide variants (SNVs) and indels (<50bp), while CoNIFER (Krumm et al. 2012) was applied to detect copy number variations (CNVs). After that, ANNOVAR (version 2015Dec14) (Wang and Hakonarson 2010) accompanied with several prediction tools were used for annotating SNVs, indels and CNVs. Notably, each variant was compared against several public databases, including Single Nucleotide Polymorphism database (dbSNP Build 147, http://www.ncbi.nlm.nih.gov/projects/SNP/snp_summary.cgi), 1000 genomes project (2015 August release, http://www.1000genomes.org/), NHLBI Exome Sequencing Project (ESP) 6500 (http://evs.gs.washington.edu/EVS/), and Exome Aggregation Consortium (ExAC release 0.3.1, http://exac.broadinstitute.org) to achieve allele frequency. In terms of possible influence on the protein function, missense variants were evaluated by several popular prediction tools, i.e., Sorting Intolerant from Tolerant (SIFT) (Ng and Henikoff 2003), Polymorphism Phenotyping version 2 (PolyPhen-2) (Adzhubei et al. 2010), MutationTaster (Schwarz et al. 2010), LRT (Chun and Fay 2009), MutationAssessor (Reva et al. 2011),

FATHMM (Gough et al. 2001), SiPhy (Garber et al. 2009), phyloP (Pollard et al. 2010), CADD (Kircher et al. 2014) and Genomic Evolutionary Rate Profiling (GERP++) (Davydov et al. 2010).

Prioritization of candidate variants and genes associated with the disease phenotype

Based on the variant annotations, a series of prioritization strategies were applied to identify candidate variants associated with the phenotypes. The detailed steps were as follows: (1) excluding variants in repeats or segmental duplications since such variants might be mainly supported by multiple mappings and had relatively low confidences; (2) excluding variants outside exonic and splicing regions; (3) excluding variants with minor allele frequency (MAF) >0.01 according to public databases; (4) excluding synonymous variants; (5) excluding non-conservative variants with score ≤ 2 according to GERP++ conservation prediction; (6) excluding variants not presenting damaging results in any protein function prediction from SIFT (D), Polyphen2 (D), MutationTaster (D) or CADD (CADD phred ≥15). Remaining data after the six steps formed a list of candidate variants and related genes. To prioritize the most likely candidate disease-causing gene, all candidate genes were then ranked by Phenolyzer (http://phenolyzer.wglab.org/)(Yang et al 2015), a tool using prior information to implicate genes involved in diseases. 'symphalangism' was input as phenotype terms into Phenolyzer.

Sanger Validation

To confirm the candidate disease-causing variant, we PCR-amplified the genomic DNA fragments in all available familial members (I-2, II-1, II-4 and III-2), then sequenced them by Sanger sequencing. The PCR primer pairs (forward 5′–CCAACTTGTGTGCCTTTCTTCCG–3′, reverse 5′–GTGGTGGAACTGGTTGGAGG–3′) were designed by Primer Z (Tsai et al 2007).

Molecular Modeling

Protein structure files (1M4U) were downloaded from the Protein Data Bank (PDB) website. Modeling was performed in UCSF Chimera version 1.10.2 following the software documentation. We used previously established procedures to perform

molecular modeling using the UCSF Chimera software, version 1.10.2 (Burger et al. 2009). Briefly, the default settings were used for all the calculations: molecular surface computation and distance measurement was performed using the built-in function of Chimera. Hydrogen bond predictions were performed with the 'FindHBond' function in 'Structural Analysis' with default parameter set-tings to relax H-bond constraints by 0.8 angstrom and 40˚. Amino acid substitution is modeled using the Rotamesr function under Structure Editing option in the Tools menu. Once the aspartic acid was substituted with a tyrosine residue, hydroten atoms were added to the tyrosine and its interacting partner, R167, in order to perform the structure minimization calculations. The tyrosine rotamer with the highest probability was chosen as the starting structure, followed by 100 steps of steepest descent for energy minimization.

Reference

Adzhubei IA, Schmidt S, Peshkin L, Ramensky VE, Gerasimova A, Bork P, et al. A method and server for predicting damaging missense mutations. Nat Methods.7(4):248-9 (2010).

Burger G, Yan Y, Javadi P, Lang BF. Group I-intron trans-splicing and mRNA editing in the mitochondria of placozoan animals. Trends Genet. 25(9):381-6(2009).

Chun S, Fay JC. Identification of deleterious mutations within three human genomes. Genome Res. 19(9):1553-61 (2009).

Davydov EV, Goode DL, Sirota M, Cooper GM, Sidow A, et al. Identifying a high fraction of the human genome to be under selective constraint using GERP++. PLoS Comput Biol. 6(12):e1001025(2010).

Garber M, Guttman M, Clamp M, Zody MC, Friedman N, Xie X.Identifying novel constrained elements by exploiting biased substitution patterns. Bioinformatics. 25(12):i54-62 (2009).

Gough J, Karplus K, Hughey R, Chothia C. Assignment of homology to genome sequences using a library of hidden Markov models that represent all proteins of known structure. J Mol Biol. 313(4):903-19(2001).

Kircher M, Witten DM, Jain P, O'Roak BJ, Cooper GM, Shendure J. A general framework for estimating the relative pathogenicity of human genetic variants. Nat Genet. 46(3):310-5 (2014).

Krumm N, Sudmant PH, Ko A, O'Roak BJ, Malig M, Coe BP, et al. Copy number variation detection and genotyping from exome sequence data. Genome Res. 22(8):1525-32(2012).

Li H, Durbin R. Fast and accurate short read alignment with Burrows-Wheeler transform. Bioinformatics. 25(14):1754-60(2009).

Li H, Handsaker B, Wysoker A, Fennell T, Ruan J, Homer N, et al. The Sequence Alignment/Map format and SAMtools. Bioinformatics. 25(16):2078-9 (2009).

Ng PC, Henikoff S. SIFT: Predicting amino acid changes that affect protein function. Nucleic Acids Res. 31(13):3812-4(2003).

Pollard KS, Hubisz MJ, Rosenbloom KR, Siepel A. Detection of nonneutral substitution rates on mammalian phylogenies. Genome Res, 20(1). 110-121(2010).

Reva B, Antipin Y, Sander, C. Predicting the functional impact of protein mutations: application to cancer genomics. Nucleic Acids Res. 39(17):e118 (2011).

Schwarz JM, Rodelsperger C, Schuelke M, Seelow D. MutationTaster evaluates disease-causing potential of sequence alterations. Nat Methods. 7(8):575-6 (2010).

Tsai MF, Lin YJ, Cheng YC, Lee KH, Huang CC, Chen YT, et al. PrimerZ: streamlined primer design for promoters, exons and human SNPs. Nucleic Acids Res. 35(Web Server issue): W63-5(2007).

Wang K, Li M, Hakonarson H. ANNOVAR: functional annotation of genetic variants from high-throughput sequencing data. Nucleic Acids Res. 38(16): e164 (2010).

Yang H, Robinson PN, Wang K. Phenolyzer: phenotype-based prioritization of candidate genes for human diseases. Nat Methods. 12(9):841-3 (2015).
